# Broadband unidirectional visible imaging using wafer-scale nano-fabrication of multi-layer diffractive optical processors

**DOI:** 10.1038/s41377-025-01971-2

**Published:** 2025-08-11

**Authors:** Che-Yung Shen, Paolo Batoni, Xilin Yang, Jingxi Li, Kun Liao, Jared Stack, Jeff Gardner, Kevin Welch, Aydogan Ozcan

**Affiliations:** 1 Electrical and Computer Engineering Department, University of California, Los Angeles, CA 90095 USA; 2 Bioengineering Department, University of California, Los Angeles, CA 90095 USA; 3 California NanoSystems Institute (CNSI), University of California, Los Angeles, CA 90095 USA; 4 Micro Optics, Optical Systems Division, Broadcom Inc., Charlotte, NC 28262 USA

**Keywords:** Optical materials and structures, Lithography, Imaging and sensing

## Abstract

We present a broadband and polarization-insensitive unidirectional imager that operates at the visible part of the spectrum, where image formation occurs in one direction, while in the opposite direction, it is blocked. This approach is enabled by deep learning-driven diffractive optical design with wafer-scale nano-fabrication using high-purity fused silica to ensure optical transparency and thermal stability. Our design achieves unidirectional imaging across three visible wavelengths (covering red, green, and blue parts of the spectrum), and we experimentally validated this broadband unidirectional imager by creating high-fidelity images in the forward direction and generating weak, distorted output patterns in the backward direction, in alignment with our numerical simulations. This work demonstrates wafer-scale production of diffractive optical processors, featuring 16 levels of nanoscale phase features distributed across two axially aligned diffractive layers for visible unidirectional imaging. This approach facilitates mass-scale production of ~0.5 billion nanoscale phase features per wafer, supporting high-throughput manufacturing of hundreds to thousands of multi-layer diffractive processors suitable for large apertures and parallel processing of multiple tasks. Beyond broadband unidirectional imaging in the visible spectrum, this study establishes a pathway for artificial-intelligence-enabled diffractive optics with versatile applications, signaling a new era in optical device functionality with industrial-level, massively scalable fabrication.

## Introduction

Deep learning has been transforming optical engineering by enabling novel approaches to the inverse design of optical systems^1–15^. For example, deep learning-driven inverse design of diffractive optical elements (DOEs) has led to the development of spatially engineered diffractive layers, forming various architectures of diffractive optical processors^16–20^ where multi-layer diffractive structures collectively execute different target functions. These diffractive processors, composed of cascaded layers with wavelength-scale features, allow precise modulation of optical fields to achieve a wide range of advanced tasks, including quantitative phase imaging^21–24^, all-optical phase conjugation^25^, image denoising^26^, spectral filtering^27–29^, and class-specific imaging^30,31^. Metasurfaces^32–34^, as another example, utilize deeply subwavelength features to achieve customized optical responses, allowing precise control over various light properties, including phase, polarization, dispersion, and orbital angular momentum. These innovations in spatially engineered surfaces represent a significant advancement in optical information processing, enabling various applications, including beam steering^35^, holography^36,37^, space-efficient optical computing^38^, and smart imaging^39,40^.

Despite the promising potential and emerging uses of diffractive optical processors and metamaterials, most of these demonstrations remain constrained to 2D implementations and longer wavelengths due to the fabrication challenges of nanoscale features in 3D diffractive architectures. Nano-fabrication techniques such as two-photon polymerization methods^9,31,41,42^ and electron beam lithography (EBL) processes^14,32,40,43,44^ have enabled the fabrication of micro- or nanoscale multi-layer diffractive designs for the near-infrared and visible spectrum. However, these designs suffer from material absorption, restricted fabrication area, limited phase bit depth for each modulation element, and 3D alignment challenges, resulting in limited degrees of freedom for more complex applications in the visible spectrum. To our knowledge, no prior demonstration reports wafer-level scalable fabrication of multi-layer diffractive surfaces operating at visible wavelengths.

**Fig. 1:**
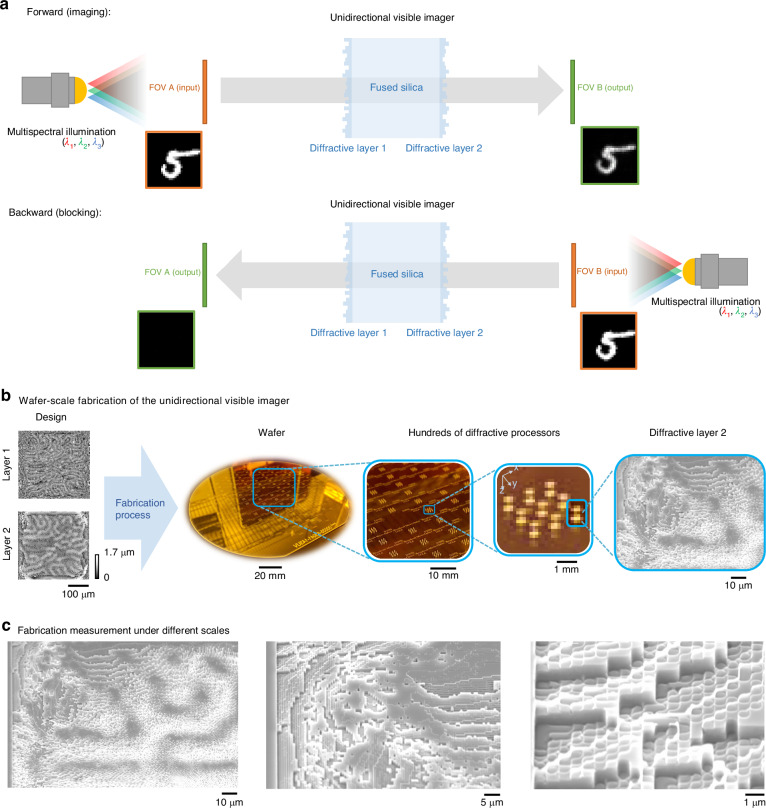
Illustration of broadband unidirectional visible imagers using wafer-scale fabrication of multi-layer diffractive optical processors. **a** A unidirectional imager reproduces the image in the forward direction (FOV A to FOV B) while blocking the image transmission in the backward direction (FOV B to FOV A). **b** Thickness profiles of the optimized diffractive layers and wafer-scale fabrication of 918 multi-layer diffractive designs on the same 6-inch wafer. **c** SEM images of the diffractive layers at different magnification factors

Here, we demonstrate multi-layer diffractive optical processors for broadband unidirectional imaging in the visible spectrum using industrial-grade on-wafer lithography^45,46^; Fig. 1. Notably, our design features the scalable fabrication of two-layer diffractive optical processors, specifically designed for the visible spectrum, achieving a 16-level phase depth per diffractive nanoscale feature. This all-optical diffractive processor enables visible image formation in only one direction—transmitting images from input field of view (FOV) A to output FOV B—while blocking and distorting image formation in the reverse direction (B→A). This work represents the first demonstration of broadband unidirectional imaging in the visible spectrum, achieved with nanoscale, polarization-insensitive, and transparent diffractive features that were optimized using deep learning.

We experimentally validated the unidirectional imaging capability of our two-layer diffractive processor across three wavelengths: 467.5 nm, 525 nm, and 627.5 nm, corresponding to blue, green, and red colors. The unidirectional imager successfully created the forward-direction images while the backward direction information was blocked and distorted for all three illumination wavelengths, well matching the numerical simulations. We also demonstrated, through both simulations and experiments, that despite being trained with three narrow spectral bands, the diffractive imager maintained successful unidirectional image transmission over a large range of wavelengths, demonstrating robustness as a broadband unidirectional imager.

Our 3D nano-fabrication method supports scalable, high-throughput manufacturing of hundreds of millions of phase features on the same wafer, making it suitable for large FOV operation over extended apertures and parallel multi-task processing. Coupled with the use of high-purity fused silica (HPFS)—highly valuable for its ultra-low energy loss and exceptional thermal stability—this advancement enables complex diffractive processing, making our multi-layer designs adaptable for a wide range of optical applications. Given that our fabrication methods overlap with the lithography processes used in semiconductor manufacturing, our diffractive optical processor designs could be monolithically integrated with other electronic or optoelectronic devices. The potential applications for diffractive unidirectional imaging using structured materials are extensive, spanning fields such as security, defense, telecommunications, and privacy protection. This study opens up new avenues for the applications of diffractive optical processors, paving the way for advanced, massively scalable solutions in intelligent imaging and sensing with visible light.

## Results

### Broadband unidirectional imager design

Figure 1a depicts a schematic of our broadband unidirectional imaging framework, illuminated by spatially coherent light across different wavelengths. This system comprises input/output FOVs and a diffractive imaging unit with two successive modulation layers that are structured transmissive surfaces. Each diffractive layer consists of 512 × 512 trainable diffractive features, with each feature having a lateral size of 714 nm and a tunable thickness, providing a phase modulation range covering 0–2π for all the desired illumination wavelengths. The two transmissive layers are made of and connected through HPFS, with both the input plane to the first diffractive layer and the second diffractive layer to the output plane interfaced via light diffraction in air; this configuration results in an axially compact system spanning ~2 mm. In addition to thermal stability, the choice of HPFS offers several other key benefits. It is mechanically strong, highly resistant to abrasion, chemically inert, and resilient to both strong acids and bases, making it suitable for harsh environments. Furthermore, HPFS is available in standard SEMI wafer form with double-sided optical polish, facilitating compatibility with wafer-scale manufacturing processes. Unlike conventional glass compositions, HPFS is a pure, amorphous form of SiO₂, allowing for precise dry etching with standard chemistries without dependence on crystal orientation (see the “Materials and methods” section for details).

The broadband unidirectional imager processes the complex fields of the multispectral input object {${{\boldsymbol{i}}}_{w}$} to produce output complex fields {${{\boldsymbol{o}}}_{w}$} at each wavelength of interest (${\lambda }_{w}$). The resulting output intensity profiles are captured in a single snapshot by a color Complementary Metal-Oxide-Semiconductor (CMOS) image sensor, providing intensity measurements ${{\boldsymbol{O}}}_{w}$. In the forward direction, the unidirectional imager faithfully reproduces the corresponding image at each wavelength within the output FOV, i.e., ${{\boldsymbol{O}}}_{w}\approx {{\alpha }_{w}\cdot {\boldsymbol{I}}}_{w}$, where ${{\boldsymbol{I}}}_{w}$ is the ground truth intensity pattern of the input object and ${\alpha }_{w}$ is a wavelength-dependent scalar constant. In the backward direction, when the input and output FOVs are reversed, the unidirectional imager blocks the image information, yielding distorted, reduced-energy patterns at the output FOV across all the desired illumination wavelengths.

Our diffractive unidirectional imaging models were optimized using error backpropagation and stochastic gradient descent-based optimization^47^, aiming to minimize a custom loss function (*L*) based on the normalized mean-squared error (NMSE), Pearson Correlation Coefficient (PCC), and diffraction efficiency between the projected intensity images and their corresponding ground truth images across all the wavelengths – calculated for both the forward and backward directions (see the “Materials and methods” section for details). To achieve broadband unidirectional imaging capability in the visible spectrum, the system was trained using wavelengths randomly sampled within {627.5 ± 10 nm, 525 ± 18 nm, 467.5 ± 7.5 nm} during each training iteration. Moreover, the training process of these broadband unidirectional imagers was adaptively balanced across different wavelengths of operation such that the asymmetrical image transmission performances of the different channels were similar to each other, without introducing a preference toward any wavelength channel, as detailed in the “Materials and methods” section. Deep learning-based training used the MNIST image dataset, and the resulting optimized diffractive layers, with 16 levels of phase for each diffractive modulation element, are shown on the left side of Fig. 1b.

**Fig. 2:**
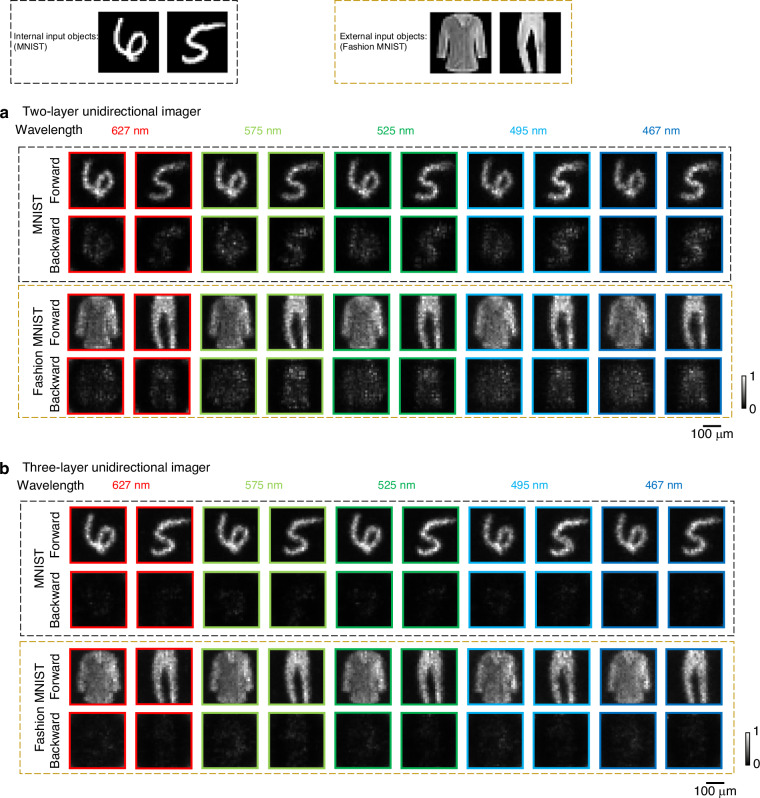
Blind testing results of broadband unidirectional visible imagers at different illumination wavelengths. **a** Diffractive output images of both the forward direction and the backward direction using the two-layer unidirectional imager design shown in Fig. 1b. **b** Diffractive output images of both the forward direction and the backward direction using the three-layer unidirectional imager design shown in Fig. S1

To evaluate the broadband imaging capability of this unidirectional imager design, we conducted a numerical analysis of its spectral response across the visible spectrum, spanning [450–650] nm with 200 uniformly sampled test wavelengths. This analysis compared the unidirectional imaging performances of two diffractive designs: (i) the two-layer unidirectional imager design (shown in Fig. 1) and (ii) a three-layer imager design that incorporates an additional diffractive layer for enhanced spectral response (see Fig. S1). The latter design, except for the number of diffractive layers, retained all the other structural parameters identical to the former two-layer configuration and utilized the same training image dataset. Here, the three-layer imager maintains the same layer-to-layer axial distance as the two-layer configuration illustrated in Fig. 1a. Consequently, the overall axial depth of the three-layer imager linearly increases compared to the original two-layer imager. To assess these broadband unidirectional imagers’ internal and external generalization capabilities, we numerically tested each design using 10,000 input images from the MNIST dataset and 10,000 input images from the Fashion MNIST dataset, both of which were never used during the training stage. Figure 2 shows examples of the output images resulting from both of these unidirectional imager designs. As illustrated in Fig. 2a, the two-layer diffractive unidirectional imager successfully reproduces forward images while significantly distorting and blocking the backward image formation, as desired. This asymmetrical image transmission remained successful across different input datasets, including the MNIST and Fashion MNIST datasets, demonstrating the unidirectional imager’s generalization capability to different types of input objects. The three-layer unidirectional imager design, shown in Fig. 2b, achieved further improved performance, not only enhancing the quality of the forward images but also further suppressing the undesired image formation in the backward direction.

**Fig. 3:**
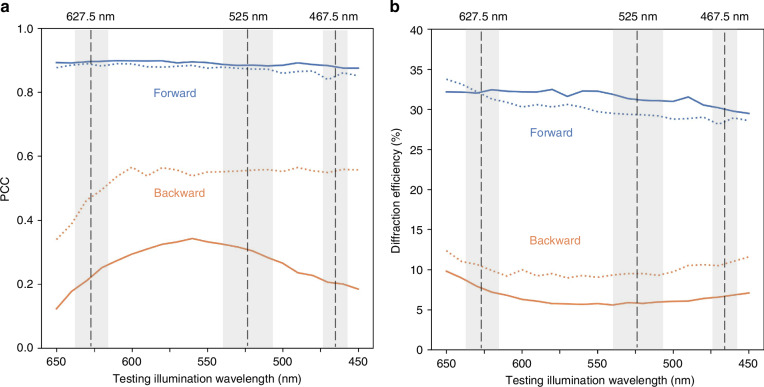
Spectral response of broadband unidirectional visible imagers. **a** Output image of PCC values as a function of the illumination wavelength for both the forward and backward directions. **b** Diffraction efficiency as a function of the illumination wavelength for both the forward and backward directions. The dashed curves represent the performance of the two-layer unidirectional imager design shown in Fig. 1b, while the solid curves represent the performance of the three-layer unidirectional imager design shown in Fig. S1. The gray areas mark the illumination wavelengths used during the training of the diffractive optical processors

Quantitative metrics reported in Fig. 3 further validated the performance of both of these diffractive unidirectional imager designs. As shown in Fig. 3a, the two-layer imager consistently maintained effective unidirectional imaging performance across different wavelengths, achieving forward PCC values of >0.86 and backward PCC values of <0.58 throughout the tested illumination spectrum, 450–650 nm. This shallower design with 2 diffractive layers also demonstrated asymmetrical energy transmission, with forward diffraction efficiency values of >28% and backward diffraction efficiency values of <13% across all the tested wavelengths, as illustrated in Fig. 3b. The deeper diffractive unidirectional imager design with 3 layers provided significant enhancements in these metrics. As shown in Fig. 3a, the forward PCC values of the deeper design with 3 optimized diffractive layers were improved to >0.89 across the entire spectrum, while the backward PCC values dropped to <0.33, demonstrating a substantial improvement in suppressing undesired image formation in the backward direction. Furthermore, Fig. 3b revealed that the forward diffraction efficiency increased to >30%, while the backward efficiency dropped to <10% across the entire test wavelength range. This increased performance asymmetry between the forward and backward directions highlights the superior capability of deeper diffractive processor designs with more degrees of freedom for enhanced unidirectional imaging.

Diffraction efficiency plays a critical role in the practical implementations of diffractive optical processors. A fundamental aspect of these designs is understanding the trade-off between output diffraction efficiency and unidirectional imaging performance. To systematically evaluate such trade-offs, we defined a figure of merit (FOM), which incorporates both the forward (imaging) and backward (blocking) performances of our system by incorporating the ratio between the forward PCC and the backward PCC, as well as the ratio between the forward and backward diffraction efficiencies, as detailed in the “Materials and methods” section. This FOM serves as a quantitative metric for assessing the overall performance of asymmetric image transmission. We trained new diffractive unidirectional imager designs with varying forward efficiency loss weights (${\alpha }_{3}$) of [0.0001, 0.0003, 0.0006, 0.0009, 0.0012] and compared the resulting FOM values and the forward diffraction efficiencies of these designs. In the same analysis, we also examined the impact of the axial depth by comparing designs with different numbers of diffractive layers (*K*)—specifically, two-layer, three-layer, and four-layer configurations. The structural parameters and the training parameters are detailed in the “Materials and methods” section. Our findings, presented in Fig. S2, reveal that increasing the forward energy penalty enhances the output diffraction efficiency while causing only a slight reduction in the unidirectional imaging performance. Additionally, the performance curve for the four-layer (*K* = 4) design consistently outperforms those of the three-layer (*K* = 3) and two-layer (*K* = 2) configurations across different efficiency weights, highlighting the benefits of increased axial depth in optimizing both the diffraction efficiency and the unidirectional imaging quality. Notably, the *K* = 4 design achieves a forward diffraction efficiency of 81.2% with an FOM value of 2.69, while the *K* = 2 design with the same loss weights has a forward diffraction efficiency of 79.4% with an FOM value of 2.23. These results demonstrate that a deeper diffractive architecture with proper energy optimization not only improves the energy transmission—directing a larger portion of the input energy forward—but also preserves favorable unidirectional imaging performance. By incorporating additional layers, it is possible to achieve a superior balance between diffraction efficiency and image quality, making multi-layer designs particularly advantageous for complex high-performance imaging applications.

To further validate the general functionality of our diffractive imager, we evaluated its performance under dynamic imaging scenarios. Specifically, we evaluated the capability to process objects placed at varying lateral shifts, orientations, and scales within the input field of view (Figs. S3–S5), simulating different imaging conditions encountered in practical applications. As demonstrated in the output examples, the diffractive imager consistently performed unidirectional imaging, maintaining forward PCC values of >0.85 and backward PCC values of <0.6 across these diverse conditions. These results highlight the robustness of our diffractive imager, demonstrating that it is not restricted to specific object types or fixed conditions but functions as a general-purpose imaging system in the forward direction while effectively blocking the image formation in the backward direction. These findings further reinforce the potential of our diffractive optical processor for a wide range of real-world imaging tasks where object position, scale, and orientation are dynamic.

### Wafer-scale fabrication of broadband unidirectional visible imagers

Figure 1b illustrates the wafer-scale fabrication of diffractive optical processors specifically designed for broadband unidirectional imaging in the visible part of the spectrum. We also highlight the zoomed-in diffractive layers and the individual nanoscale diffractive features in Fig. 1b. Using wafer-scale manufacturing, we fabricated 918 multi-layer diffractive designs on a single wafer, exhibiting ~0.5 billion 16-phase-level diffractive features at the nanoscale.

**Fig. 4:**
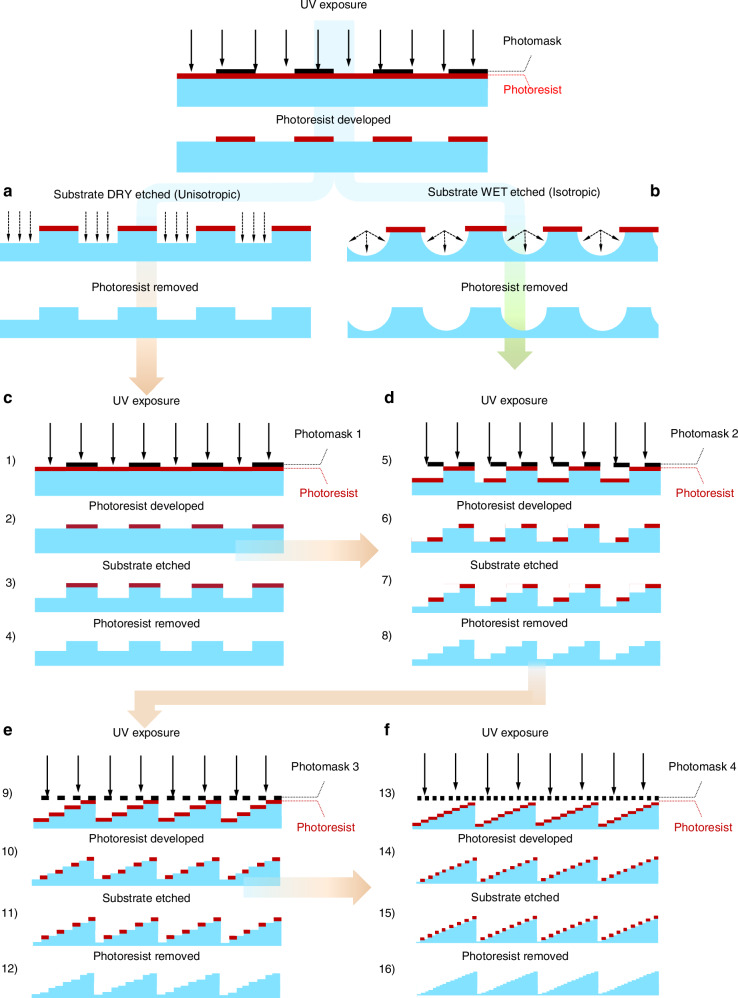
Lithography process for the fabrication of diffractive optical layers. **a** Illustration of dry etching versus **b** wet etching. **c** Fabrication sequence to obtain a 2-level diffractive surface. **d** Subsequent fabrication to generate a 4-level diffractive surface. **e** Subsequent fabrication to generate an 8-level diffractive surface. **f** Subsequent fabrication to generate a 16-level diffractive surface. **c**–**f** Depict a conceptual fabrication of a 16-level diffractive surface designed using the binary optics model (2 N)

To elaborate on the fabrication approach of our diffractive unidirectional visible imager, we first illustrate a generic manufacturing process, as shown in Fig. 4, which is used to create the binary architecture that characterizes DOEs. This structure is composed of discrete surface-relief micro and nanoscale “pixels”^48^, and the manufacturing process behind it is closely related to the lithographic processes used to fabricate semiconductors at the wafer scale. First, a substrate wafer is coated with a thin, uniform layer of photoresist using a spin-coater. The spin-coating process is critical because the resulting layer of photoresist—typically a few hundred nanometers to several micrometers thick—directly influences all subsequent photolithographic and etching steps. Another key element in our fabrication process is the use of photomasks, also known as reticles. To elaborate more, a photomask is a plate made of fused silica or quartz, typically 6 inches square, covered with a pattern of opaque, transparent, and/or phase-shifting areas. The function of these areas of the photomask is to either block, partially transmit, or fully transmit the UV radiation, so that a specific pattern can be projected into a layer of photoresist. A photomask is made by means of projection-exposing or direct-writing a given pattern onto a resist-coated chrome mask blank. The image is transferred permanently in the chrome film by means of etching after the developed resist is removed. Importantly, photomasks are not in direct contact with the photoresist. Instead, they serve as the image source in an optical system—using UV steppers or scanners—to project a reduced image (typically at 4× or 5× reduction) onto the wafer. Depending on the desired minimum size, the photoresist may be patterned with a photomask using either direct (1×) contact printing or a projection process such as a stepper or scanner (4×–5×). The (1×) or (4×–5×) symbols describe the reduction ratio of the image that is projected from the photomask onto the layer of the photoresist on the wafer. For example, if a given feature on a photomask has a physical dimension of 20 µm, that feature will be projected onto the photoresist as a feature having a physical dimension of 5 µm under 4×.

In this work, we utilized projection lithography to fabricate our diffractive design since the minimum feature size obtainable using projection lithography (~100 nm) is significantly smaller than that achievable using contact printing (~1 µm). After exposure using a suitable photomask, the resist is developed and fully cured. A permanent pattern on the substrate’s surface is then made through etching, which removes the exposed areas of the wafer. As an irreversible step, etching is the most critical phase of the process.

For manufacturing distinct geometries and vertical sidewalls, dry etching^49^, typically using gas-phase chemical reactions, is preferred over wet etching, which uses amorphous materials with liquid chemistry. As shown in Fig. 4a, the advantage of dry etching is that it can be made unidirectional, meaning that etching takes place at a high etch rate in one direction, typically in a vertical direction with respect to the surface of the substrate wafer. This dry etching method preserves the lateral size and shape of features. On the contrary, as shown in Fig. 4b, wet etching proceeds with the same etch rate in every direction (omnidirectional). This isotropy typically causes broadening of feature dimensions and rounding of edges, limiting the precision of fabricated structures. Given these characteristics, our fabrication process employed chlorine-based dry etching chemistries to achieve precisely controlled axial depths. This approach enabled us to produce vertical profiles without altering the diffractive elements’ lateral dimensions, ensuring each feature’s structural integrity and optical performance.

We achieved the fabrication of 16-phase-level diffractive structures on two opposing surfaces of our 6-inch HPFS wafer (see the “Materials and methods” section). This type of architecture can be generalized by repeating a 2-level design concept. In this approach, the fabrication involves repeating a cycle of coating, exposing, developing, and etching multiple times with a customized photomask for each level. Figure 4c–f illustrates this process, showing how a blazed structure can be approximated by successively etching smaller steps with precise relative alignment.

Each step size is controlled solely by the etching time, provided that the etching rate under specific operating conditions is known. Patterning of the photoresist is achieved using 365 nm UV radiation, provided by an i-Line UV Stepper and different photomasks through a projection process. By repeating the fabrication cycle four times with four different photomasks, 16-phase-level diffractive surfaces were successfully fabricated on the wafer. Each photomask projection is carefully aligned to compose a complex image through a series of sequential exposures onto the photoresist layer. Precise alignment is ensured using specially designed alignment marks, which are imprinted onto the substrate surface as a reference layer and incorporated into each additional layer. These alignment marks serve as spatial references for subsequent exposures, allowing for highly accurate layer positioning. They can take the form of precision gratings—patterns composed of vertically sided lines perpendicular to the substrate surface—or they may be embedded within other patterns to serve as known image references. Custom alignment sensors detect these gratings or special marks to determine the exact spatial location of the substrate, ensuring consistency and precision across multiple fabrication cycles.

Figure 1b, c provides a detailed look at both the numerically optimized diffractive thickness profiles and the experimentally fabricated layers. Figure 1b shows the wafer layout, with close to a thousand identical diffractive optical processor designs arranged in a 2D array on the same 6-inch wafer. High-resolution measurements obtained via scanning electron microscopy (SEM) (Fig. 1c) and confocal microscopy (Fig. S6) reveal nanoscale accuracy in both the axial depth precision and lateral resolution. To evaluate the quantitative precision of the fabrication, etch depth error was measured on test structures across the wafer according to Six Sigma and International Organization for Standardization (ISO) standards^50,51^. Our unidirectional imager fabrication achieved an etch depth error within 3–5% across all diffractive designs across different wafers, ensuring consistent performance.

Our fabrication approach for multi-layer diffractive optical processors surpasses other methods, such as two-photon polymerization^9,31,42,52^, optical Fourier surfaces^43,44^, resin stamping^53^, and mask-less grayscale exposure^54,55^, in several critical aspects. It supports a significantly larger lateral area, improved axial resolution, and a phase depth of 16 levels, all while enabling wafer-level multi-layer fabrication. As shown in Table 1, state-of-the-art two-photon polymerization-based 3D printing demonstrated the fabrication of multi-layer diffractive processors with a lateral feature size of ~400 nm and a minimum axial step size of ~10 nm, but this was limited to a total lateral size of <100 μm, restricting its applications. Similarly, the fabrication of optical surfaces using EBL demonstrated single-layer diffractive surfaces with a minimum axial step size of ~20 nm, but it was also limited to a total lateral size of <100 μm. Another fabrication approach using resin stamping demonstrated single-layer diffractive surfaces with a lateral feature size of 4 μm and an axial step size of ~160 nm, providing 8 thickness levels. Mask-less grayscale exposure, on the other hand, has been used to fabricate a two-layer diffractive processor with a lateral feature size of 3 μm and an axial step size of ~125 nm. In contrast, our method demonstrated a lateral feature size of 714 nm, an axial step size of ~100 nm, and 16-phase levels per diffractive feature. Moreover, it supports wafer-level multi-layer fabrication, accommodating ~0.5 billion nanoscale phase features per wafer. This capability ensures enhanced repeatability, precise fabrication consistency, and scalability for mass production, setting a new benchmark for visible diffractive optical processors. Therefore, these combined advantages support more complex and precise designs than previously possible.

**Table 1:** Comparison of nano-fabrication methods for diffractive optical processors

Methods	Maximum lateral size (μm)	Lateral feature size (nm)	Axial step size (nm*)*	Number of phase levels per feature	Number of independent phase features per chip	Wafer-level multi-layer fabrication
Two-photon polymerization^9,31,42,52^	<100	~300–400	~10–300	>100	<0.1 million	No
Optical Fourier surfaces^43,44^	<200	~20–400	~20	>100	<1 million	No
Resin stamping^53^	>1000	4000	~160	8	<5 million	No
Mask-less grayscale exposure^54,55^	>1000	3000	~125	16	<4 million	No
This work	>1000	714	~100	16	~0.5 billion	Yes

### Experimental demonstration of a broadband unidirectional visible imager

**Fig. 5:**
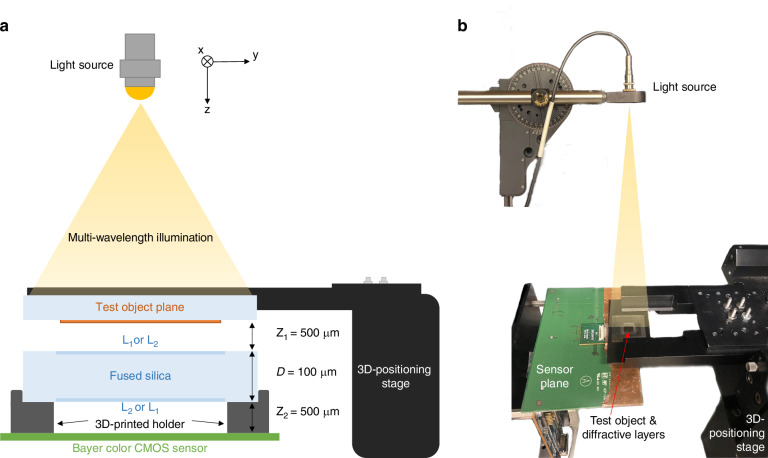
Experimental setup of broadband unidirectional visible imaging. **a** Illustration of the experimental setup, including the tunable laser source, the test object, the fabricated diffractive layers, and the image sensor. **b** A photo of the experimental setup

To experimentally validate our fabricated broadband unidirectional imager, we implemented a setup comprising a tunable laser source, an array of test objects (never used in training), the fabricated multi-layer diffractive design, and a color CMOS image sensor, as illustrated in Fig. 5a. We utilized a supercontinuum light source to achieve multispectral illumination, with each spectral band filtered down to ~5 nm bandwidth by an acousto-optic tunable filter. In our experiments, we utilized three narrow spectral lines (~467.5 nm, ~525 nm, and ~627.5 nm) to create multi-color illumination. For the test objects, 100 binary transmittance patterns from the MNIST dataset were fabricated using EBL (see the “Materials and methods” section). For precise alignment and multi-object imaging, we employed a 3D positioning stage to adjust the x-y-z position of the sample. A 3D-printed holder maintained a 500 μm separation between the diffractive layer and the sensor, and a rotational stage ensured accurate vertical alignment of the laser beam to the sensor. Figure 5b shows a photograph of the assembled experimental setup.

**Fig. 6:**
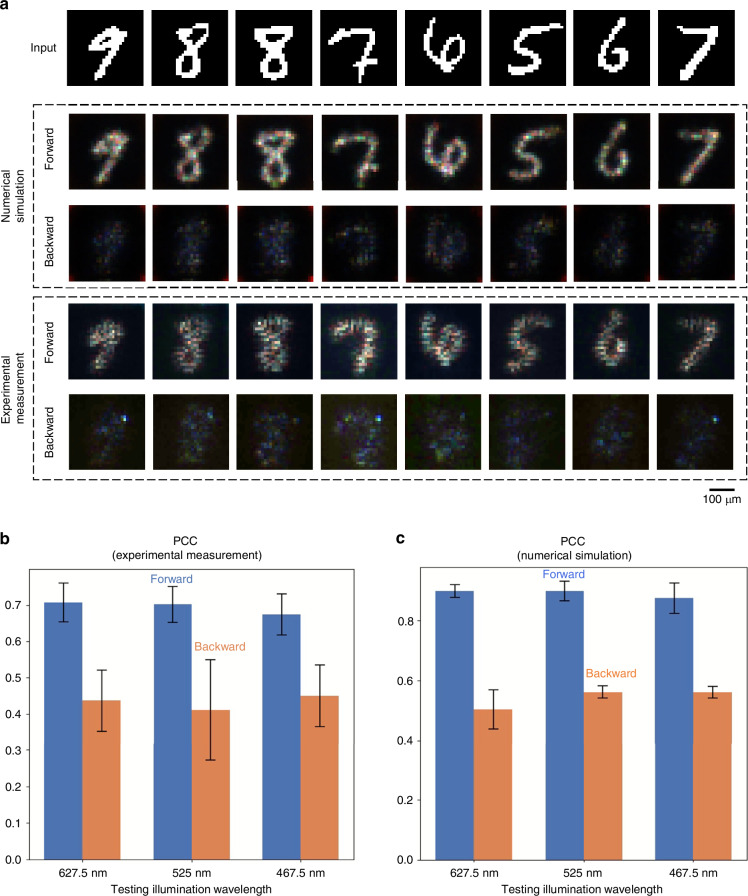
Experimental results of broadband unidirectional visible imaging. **a** Experimentally measured diffractive outputs of both the forward and the backward directions, along with the numerically simulated outputs. **b** Output image PCC values of the forward and the backward directions from the experimental measurements at three illumination wavelengths. **c** Output image PCC values of the forward and the backward directions from the numerical simulations at three illumination wavelengths. These values are calculated across test output images, reported with the mean values and the standard deviations shown as error bars

As shown in Fig. 6a, the experimental measurements aligned well with our simulation results. The input patterns in the forward direction were successfully reproduced across the three illumination wavelengths, while the input patterns in the backward direction were significantly suppressed and distorted, as desired. Quantitative performance evaluations using the PCC metric, shown in Fig. 6b, c, yielded a forward average PCC of 0.889 ± 0.036 and a backward average PCC of 0.546 ± 0.041 in simulations, compared to a forward average PCC of 0.694 ± 0.052 and a backward average PCC of 0.435 ± 0.094 in our experiments across the three tested wavelengths.

To further validate the broadband capability of our unidirectional imager, we conducted additional experiments using various wavelengths spanning the visible spectrum: [627.5 nm, 600 nm, 575 nm, 550 nm, 525 nm, 510 nm, 490 nm, and 467.5 nm]. As shown in the output image visualizations in Fig. S7, the diffractive unidirectional imager consistently achieved nearly identical asymmetrical image transmission across all these illumination wavelengths. These experimental results further confirmed that the diffractive design could successfully perform unidirectional imaging across a broad range of wavelengths within the visible spectrum, further establishing the effectiveness of its broadband operation.

**Fig. 7:**
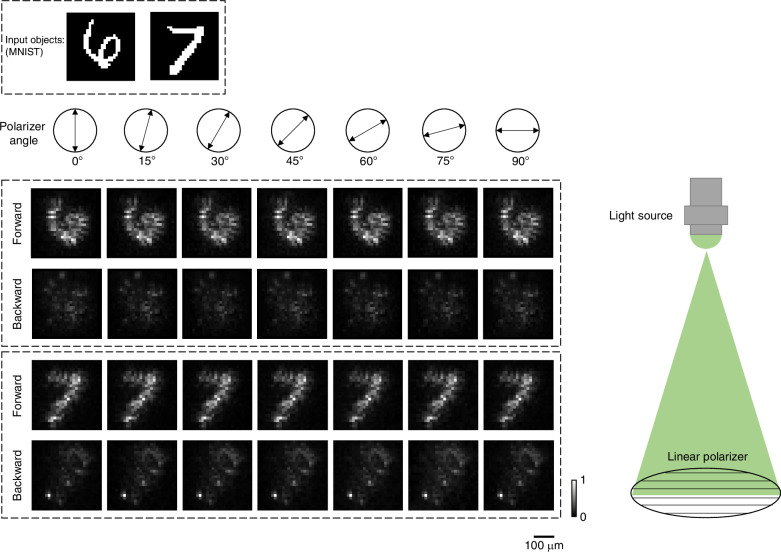
Experimental results of unidirectional visible imaging under different polarization states. Experimentally measured diffractive output images of both the forward and the backward directions at different angles of the linear polarizer, placed in front of the illumination light source

Additionally, our unidirectional imager inherently maintains its functionality regardless of the polarization state of the input light, owing to the use of HPFS. To experimentally validate the model’s polarization insensitivity, we conducted unidirectional imaging experiments using the same setup as shown in Fig. 5, with the addition of a linear polarizer placed between the light source and the input object. In these experiments, we measured the output images while varying the polarizer’s orientation at different angles in a range of [0°, 15°, 30°, 45°, 60°, 75°, 90°]. Here, the operational wavelength of the input illumination was set as 525 nm. As shown in Fig. 7, the forward-direction output images consistently maintained high quality across all the polarization angles, while the image transmission in the backward direction was effectively suppressed. These results confirm the polarization-insensitive nature of our unidirectional imager, ensuring reliable and effective performance across varying polarization states.

## Discussion

In this work, we employed unidirectional imaging as a testbed to illustrate the visible design and functionality of wafer-scale diffractive optical processors with approximately 0.5 billion phase features fabricated on the same 6-inch wafer with 16 levels of phase per feature. While the diffractive optical processors presented in this work are inherently reciprocal, the unidirectional imaging effect is achieved through task-specific engineering of the system’s asymmetrical forward and backward point spread functions (PSFs)^56,57^. By designing spatially varying PSFs using a lossy, reciprocal optical system, our unidirectional imager designs ensure that an object produces a clear image in the forward direction while yielding a distorted and suppressed output when the illumination light travels in the reverse direction. Furthermore, we want to highlight that each dielectric feature in this wavelength-multiplexed diffractive processor is based on material thickness variations, allowing it to simultaneously modulate all wavelengths within the spectrum of interest. As a result, each wavelength channel exhibits a different error gradient with respect to its assigned optical transformation. Consequently, the optimization of the diffractive layers across multiple illumination wavelengths deviates from the ideal path of an individual wavelength, enabling enhanced broadband unidirectional imaging performance. Importantly, the demonstrated system can be seamlessly adapted to a variety of applications, including unidirectional image magnification^58^, classification^42^, and operations under incoherent or partially coherent illumination^57,59^.

A critical factor for realizing a practical diffractive optical processor is controlling the misalignments between the input/output FOVs and the diffractive layers in a 3D topology. Alignment marks on sequential layers placed on separate surfaces differ significantly from aligning photomasks on a single surface. Achieving double-sided alignment—essential for fabricating a double-sided diffractive imager—involves a more complex and demanding process. This can be accomplished either by imaging the alignment process through the substrate material or by using views from opposite sides, while carefully mitigating parallax effects and tool-induced shifts during both the initial exposure and the subsequent metrology validation. Typically, custom-designed alignment marks include patterns composed of precision gratings—lines with vertical sides that are perpendicular to the substrate surface—or other marks integrated into patterns to create a known reference image. Custom alignment sensors utilize these gratings or specialized marks to determine the alignment of layers. Using these specialized alignment marks in our fabrication process, we reduced lateral misalignments between the top and bottom surfaces to <3 μm. Although the residual misalignment is small, it could still affect the phase modulation accuracy in our broadband unidirectional imager. The performance degradation from such misalignments is mitigated by applying a vaccination strategy^18,25,31^. To “vaccinate” a diffractive optical processor, random 3D misalignments are modeled in the optical forward model during the training stage so that the diffractive processor learns to adapt to these imperfections. This approach has been proven to significantly improve the robustness of diffractive optical processors against misalignments and fabrication imperfections, and the same strategy was applied to the experimental demonstration of broadband unidirectional imagers reported in Figs. 1 and 6 (see the “Materials and methods” section for details). Furthermore, fabrication imperfections such as thickness errors or pixel gaps/walls can also degrade the imaging performance of our diffractive imagers. By incorporating random pixel errors and gap variations into the optimization process—a vaccination strategy similar to that described above—the negative impact of these imperfections can be mitigated.

One of the key advantages of our fabrication method is that it allows our diffractive processor designs to integrate seamlessly with electronic components by utilizing silicon photonics^60,61^ to minimize alignment and thermal management challenges. Such an on-chip integrated approach not only enhances performance but also reduces the complexity and footprint of the hybrid system.

In conclusion, we developed a broadband diffractive unidirectional imaging framework that works in the visible spectrum, utilizing HPFS for its exceptional transparency and thermal stability. This system demonstrates robustness across a broad wavelength range and maintains alignment tolerance. Our wafer-level nano-fabrication technique for the creation of diffractive optical processors enables scalability to large FOV applications and multi-tasking; it also allows for seamless integration with electronic components such as CMOS image sensors. This diffractive imaging framework holds significant promise to advance intelligent imaging and sensing applications in the visible spectrum using passive optical elements.

## Materials and methods

### Optical forward model of a broadband unidirectional visible imager

Our diffractive unidirectional imager design consists of $K$ consecutive diffractive layers, each containing thousands of precisely positioned diffractive features. In the numerical forward model, these layers are treated as thin planar structures that modulate incident coherent light with complex transmission functions. For any given $s$ th diffractive feature on the $l$ th layer located at $({x}_{s},{y}_{s},{z}_{l})$, its complex-valued transmission coefficient, dependent on the material thickness value ${h}_{s}^{l}$, can be expressed by the following equation:

1 $$\begin{array}{c}t\left({x}_{s},{y}_{s},{z}_{l};\lambda \right)=\exp \left(\frac{-2\pi \kappa {\left(\lambda \right)h}_{s}^{l}}{\lambda }\right)\exp \left(\frac{-j2\pi \left(n\left(\lambda \right)-{n}_{{\rm{air}}}\right){h}_{s}^{l}}{\lambda }\right)\end{array}$$

Here, $n\left(\lambda \right)$ and $\kappa \left(\lambda \right)$ are the real and imaginary components of the material’s complex refractive index $\widetilde{n}\left(\lambda \right)$, i.e., $\widetilde{n}\left(\lambda \right)=n\left(\lambda \right)+j\kappa \left(\lambda \right)$. For all the diffractive unidirectional imaging designs reported in this paper, we selected HPFS as the material of the diffractive layers^62^, with the refractive index curve $n\left(\lambda \right)$ provided in Fig. S8. As HPFS exhibits negligible absorption in the visible range, $\kappa \left(\lambda \right)$ was assumed to be 0. The thickness value $h$ of each diffractive element is composed of two parts: a learnable thickness ${h}_{{\rm{learnable}}}$ and a base thickness ${h}_{{\rm{base}}}$, such that:

2 $$\begin{array}{c}h={h}_{{\rm{learnable}}}+{h}_{{\rm{base}}}\end{array}$$

Here, ${h}_{{\rm{learnable}}}$ is the tunable thickness value of each diffractive feature optimized during the training process and is constrained within the range [0, ${h}_{\max }$]; ${h}_{{\rm{base}}}$ is a fixed value representing the base thickness that acts as the substrate support for the diffractive features, which was empirically chosen as 200 nm. In this paper, ${h}_{\max }$ was set as 1649.5 nm, corresponding to a full phase modulation range from 0 to 2π for the longest wavelength of interest (${\lambda }_{1}$). Here, the trainable thickness quantization is set to 16 levels based on the fabrication constraint.

To numerically model free-space propagation of coherent light between the diffractive layers, we employed the Rayleigh-Sommerfeld scalar diffraction theory. Each ${s}^{{\rm{th}}}$ diffractive feature on the ${l}^{{\rm{th}}}$ layer at $({x}_{s},{y}_{s},{z}_{l})$ is defined as the source of a secondary wave, generating a complex field at wavelength $\lambda$ given by the equation:

3 $$\begin{array}{c}{w}_{s}^{l}\left(x,y,z;\lambda \right)=\frac{z-{z}_{l}}{{\left({r}_{s}^{l}\right)}^{2}}\,\left(\frac{1}{2\pi {r}_{s}^{l}}+\frac{n}{j\lambda }\right)\exp \left(\frac{j2\pi n{r}_{s}^{l}}{\lambda }\right)\end{array}$$

Here, ${r}_{s}^{l}=\sqrt{{\left(x-{x}_{s}\right)}^{2}+{\left(y-{y}_{s}\right)}^{2}+{\left(z-{z}_{l}\right)}^{2}}$. These secondary waves propagate to the next layer (the ($l$+1)th layer), and the optical field that reaches the $p$ th diffractive feature in the ($l$ +1)th layer, located at $({x}_{p},{y}_{p},{z}_{l+1})$, can be computed by the convolution of the complex amplitude ${u}_{s}^{l}$ from the previous layer with the impulse response function ${w}_{s}^{l}({x}_{p},{y}_{p},{z}_{l+1};\lambda )$. The resulting field is then modulated by the transmission function $t({x}_{p},{y}_{p},{z}_{l+1};\lambda )$ of the ($l$+1)th diffractive layer, which can be expressed as:

4 $$\begin{array}{c}{u}_{p}^{l+1}\left(x,y,z;\lambda \right)=t\left({x}_{p},{y}_{p},{z}_{p};\lambda \right)\cdot\mathop{\sum}\limits _{s}{u}_{s}^{l}{w}_{s}^{l}\left({x}_{p},{y}_{p},{z}_{l+1};\lambda \right)\end{array}$$

For the diffractive unidirectional visible imaging designs reported in Fig. 1 and Fig. S1, the sizes of the input FOV and the output FOV were both set to be 360 × 360 μm. The input/output FOV consists of *N*_*x*_ × $N$_*y*_ = 28 × 28 pixels, resulting in each output pixel having a size of 12.85 μm × 12.85 μm. To achieve the unidirectional imaging task, we designed the diffractive imager to possess 512 × 512 diffractive features per layer. As the diffractive feature has a size of 714 nm, each layer has a total size of ~366 × 366 μm. The axial distance between the diffractive layers was all set to 1000 μm, while the axial distances from the input plane to the first diffractive layer and from the last diffractive layer to the output plane were both set to 500 μm. For the diffractive unidirectional visible imager designs reported in Fig. S2, the sizes of the input FOV and the output FOV were both set to be 180 × 180 μm. The input/output FOV consists of ${N}_{x}$ × ${N}_{y}$ = 14 × 14 pixels, resulting in each output pixel having a size of 12.85 μm × 12.85 μm. To achieve the unidirectional imaging task, we designed the diffractive imager to possess 256 × 256 diffractive features per layer. As each diffractive feature has a lateral size of 714 nm, each diffractive layer has a total size of ~183 × 183 μm. The axial distance between the diffractive layers was set to 500 μm, while the axial distances from the input plane to the first diffractive layer and from the last diffractive layer to the output plane were both set to 250μm.

In the numerical simulations of all the diffractive unidirectional imaging designs, the spatial sampling period of the simulated complex fields was set to 238 nm. A spatial sampling period of 238 nm (~λ/2 for visible wavelengths) is chosen to ensure accurate numerical modeling of the propagating optical fields. With this half-wavelength spatial sampling, the angular spectrum method was used to precisely model the free-space wave propagation between the diffractive planes, which are axially separated by hundreds of wavelengths. Additionally, during training, we introduced random axial shifts within a range of [−20, 20] μm and random lateral shifts within a range of [−3, 3] μm between the layers. These perturbations were implemented to mitigate the impact of experimental fabrication imperfections and misalignments.

### Training loss functions and performance evaluation metrics

The broadband unidirectional imagers presented in this work are designed to closely align the forward output intensity ${{\boldsymbol{O}}}_{F,w}$ with the input/target intensity profile, ${{\boldsymbol{I}}}_{w}$, while suppressing the backward output intensity, ${{\boldsymbol{O}}}_{B,w}$, at each wavelength channel of interest, ${\lambda }_{w}$. To achieve this, we defined a loss function ${{\mathcal{L}}}_{w}$ for each wavelength channel that combines several loss terms, i.e.,:

5 $$\begin{array}{c}{{\mathcal{L}}}_{w}={{\rm{\alpha }}}_{1}{\rm{NMSE}}\left({{\boldsymbol{O}}}_{F,w},{{\boldsymbol{I}}}_{w}\right)+{{\rm{\alpha }}}_{2}{\rm{PCC}}\left({{\boldsymbol{O}}}_{B,w},{{\boldsymbol{I}}}_{w}\right)+\,{{\rm{\alpha }}}_{3}\exp \left(-\eta \left({{\boldsymbol{O}}}_{F,w},{{\boldsymbol{I}}}_{w}\right)\right)+{{\rm{\alpha }}}_{4}\eta \left({{\boldsymbol{O}}}_{B,w},{{\boldsymbol{I}}}_{w}\right)\end{array}$$

In the first term, NMSE penalizes the structural differences between the forward output intensity ${{\boldsymbol{O}}}_{F,w}$ and the target intensity ${{\boldsymbol{I}}}_{w}$, defined as:

6 $$\begin{array}{c}{\rm{NMSE}}({{\boldsymbol{O}}}_{F,w},{{\boldsymbol{I}}}_{w})=\displaystyle\frac{1}{{N}_{x}{N}_{y}{\sum }_{x,y}{\left|{I}_{w}\left(x,y\right)\right|}^{2}}\mathop{\sum }\limits_{{\rm{x}},{\rm{y}}}{|{I}_{w}\left(x,y\right)-{{\rm{\sigma }}}_{w}{O}_{F,w}\left(x,y\right)|}^{2}\end{array}$$

Here, the term ${N}_{x}\times {N}_{y}$ refers to the number of pixels at the input or output FOV. ${{\rm{\sigma }}}_{w}$ is a scalar used to normalize the forward output intensity, ensuring that the ${\rm{NMSE}}$ is not affected by the output diffraction efficiency. This constant ${{\rm{\sigma }}}_{w}$ is computed as:

7 $$\begin{array}{c}{{\rm{\sigma }}}_{w}=\,\frac{{\sum }_{x,y}{I}_{w}\left(x,y\right){O}_{F,w}\left(x,y\right)}{{{\sum }_{x,y}|{O}_{F,w}\left(x,y\right)|}^{2}}\end{array}$$

The PCC in the second term measures the linear correlation between ${I}_{w}$ and the backward output intensity ${{\boldsymbol{O}}}_{B,w}$, range from −1 and 1; it is calculated by:

8 $$\begin{array}{c}{\rm{PCC}}\left({{\boldsymbol{O}}}_{B,w},{{\boldsymbol{I}}}_{w}\right)=\frac{\sum \left({{\boldsymbol{I}}}_{w}-\,\bar{{I}_{w}}\right)\left({{\boldsymbol{O}}}_{B,w}-\,\bar{{{\rm{O}}}_{B,w}}\right)}{\sqrt{\sum {\left({{\boldsymbol{I}}}_{w}-\bar{{I}_{w}}\right)}^{2}\sum {\left({{\boldsymbol{O}}}_{B,w}-\bar{{{\rm{O}}}_{B,w}}\right)}^{2}}}\end{array}$$

Here, $\bar{{I}_{w}}$ and $\bar{{{\rm{O}}}_{B,w}}$ are the mean values of ${{\boldsymbol{I}}}_{w}$ and ${{\boldsymbol{O}}}_{B,w}$, respectively. This term ensures the backward output intensity is distorted and has a minimized correlation to the input object.

The third term in Eq. (5) is a diffraction efficiency-related loss function that boosts the forward diffraction efficiency^22,25,63^, $\eta \left({{\boldsymbol{O}}}_{F,w},{{\boldsymbol{I}}}_{w}\right)$, defined as:

9 $$\begin{array}{c}\eta \left({{\boldsymbol{O}}}_{F,w},{{\boldsymbol{I}}}_{w}\right)=\frac{{\sum }_{x,y}{O}_{F,w}\left(x,y\right)}{{\sum }_{x,y}{I}_{w}\left(x,y\right)}\end{array}$$

The fourth term in Eq. (5), in contrast to the third term, is a diffraction efficiency-reduced loss function that minimizes the backward diffraction efficiency $\eta \left({{\boldsymbol{O}}}_{B,w},{{\boldsymbol{I}}}_{w}\right)$. For the unidirectional imager designs shown in Fig. 1 and Fig. S1, the weights ${{\rm{\alpha }}}_{1}$, ${{\rm{\alpha }}}_{2}$, ${{\rm{\alpha }}}_{3}$ and ${{\rm{\alpha }}}_{4}$ were empirically set to 1.0, 0.00003, 0.0003, and 0.001, respectively. For the unidirectional imager designs analyzed in Fig. S2, the values of ${{\rm{\alpha }}}_{3}$ were set as 0.0001, 0.0003, 0.0006, 0.0009, and 0.0012.

In the training of a unidirectional imager, the total loss is calculated by averaging the loss values across all wavelength channels to optimize different wavelengths simultaneously. The overall loss function is therefore derived as:

10 $$\begin{array}{c}{{\mathcal{L}}}_{{\rm{total}}}=\frac{1}{{N}_{w}}\mathop{\sum }\limits_{w=1}^{{N}_{w}}{{{\upbeta }}}_{w}{{\mathcal{L}}}_{w}\end{array}$$

Here, ${{\rm{\beta }}}_{w}$ represents the dynamic channel balance weight for each ${{\mathcal{L}}}_{w}$, which adjusts the performance balance across different wavelength channels during the optimization^64^. Initially set as 1, ${{\rm{\beta }}}_{w}$ is adaptively updated during each training iteration according to the formula:

11 $$\begin{array}{c}{{{\upbeta }}}_{w}\leftarrow \max \left(0.01\times \left({{\mathcal{L}}}_{w}-{{\mathcal{L}}}_{{mean}}\right)+{{{\upbeta }}}_{w},\,0\right)\end{array}$$

Here, ${{\mathcal{L}}}_{{mean}}$ represents the average of the loss values from different wavelength channels.

To evaluate the performance of each unidirectional diffractive imager, we also utilized the PCC metric, as defined in Eq. (8), to assess the linear correlation between the forward (or backward) outputs and the ground truth images across different wavelength channels. Based on empirical evidence and previous studies^23,24,30,56^, a PCC value that exceeds, e.g., 0.80–0.85 indicates high image fidelity for imaging applications.

In addition, we defined the FOM of the unidirectional diffractive imager to quantify the overall unidirectional imaging performance, which was calculated as:

12 $$\begin{array}{c}{{\rm{FOM}}}_{w}\left({{\boldsymbol{O}}}_{F,w},{{\boldsymbol{O}}}_{B,w},{{\boldsymbol{I}}}_{w}\right)=\frac{{\rm{PCC}}\left({{\boldsymbol{O}}}_{F,w},{{\boldsymbol{I}}}_{w}\right)}{{\rm{PCC}}\left({{\boldsymbol{O}}}_{B,w},{{\boldsymbol{I}}}_{w}\right)}+0.1\times \frac{\eta \left({{\boldsymbol{O}}}_{F,w},{{\boldsymbol{I}}}_{w}\right)}{\eta \left({{\boldsymbol{O}}}_{B,w},{{\boldsymbol{I}}}_{w}\right)}\end{array}$$

### Training data preparation and other implementation details

To optimize the diffractive models presented in this study, we utilized a training dataset consisting of 55,000 images from the MNIST handwritten digits. An image augmentation strategy was employed during training to improve the models’ generalization capabilities. This strategy included random translation and flipping operations (up-down and left-right) applied to the input images, implemented using the RandomAffine function in PyTorch. The translation range was uniformly sampled within [−5, 5] pixels. Additionally, each flipping operation was performed with a probability of 0.5.

All the diffractive unidirectional imager models used in this work were trained using PyTorch (version 2.5.0, Meta Platform Inc.). We selected the AdamW optimizer^65^, and its parameters were taken as the default values and kept identical in each model. The batch size was set as 8. The learning rate, starting from an initial value of 0.03, was set to decay at a rate of 0.5 every 10 epochs, respectively. The training of the diffractive models was performed with 20 epochs. For the training of our diffractive models, we used a workstation with a GeForce GTX 3090Ti graphical processing unit (Nvidia Inc.) and a Core i9-11900 central processing unit (Intel Inc.) and 128 GB of RAM, running on Windows 10 operating system (Microsoft Inc.). The typical time required for training a diffractive broadband unidirectional visible imager is ~32 h.

### Nano-fabrication details

The unidirectional visible imager design shown in Fig. 1b is fabricated on a 6-inch HPFS substrate. This device features a dual-sided diffractive design, with two diffractive layers patterned on opposite surfaces of the substrate. Each diffractive feature in these layers has 16 discrete phase levels, achieving a 4-bit depth phase to cover the 0–2π range at all target illumination wavelengths in the visible spectrum.

To ensure precise alignment across multiple processing steps, specialized micro- and nanoscale alignment marks were patterned on the wafer surfaces. These marks serve as reference points for lithography tools, enabling highly accurate alignment of sequential layers. Even when the wafer is moved, processed, and reloaded, these alignment marks ensure that maximum misalignment tolerance is minimized to 3 µm between the top and bottom devices, thus achieving the high precision required for the design.

HPFS was selected as the core material for fabricating our diffractive unidirectional imager due to its extraordinarily low coefficient of thermal expansion (CTE), as shown in Table 2. This property ensures that even under significant temperature changes, the dimensions and structural integrity of the diffractive features remain stable, preserving the desired optical output without deformation, expansion, or shrinkage. Since the minimum feature size of the diffractive structures is ~0.7 µm, which exceeds the operational wavelengths, polarization effects induced by surface nanostructures can be ignored. This allows the interaction between the input light and the diffractive layers to be accurately modeled through the phase patterns of each diffractive layer.

**Table 2:** Optical materials for DOEs

Materials	CTE (ppm/°C)	Spectral Range (nm)	*n*	Note(s)
High-purity fused silica^62^	0.55	150–3000	~ 1.5	High transparency and extremely low CTE. Thermally stable and chemically inert. High strength and scratch resistance.
Silicon (Si)^66^	2.6	1000–7000	~ 3.5	Good for IR applications. Moderate CTE. High *n* translates into tight tolerances for fabrication (etch depth).
Germanium (Ge)^66^	5.8	2000–20,000	~ 4.0	Good for mid-IR applications. Extensively used as a substrate for the production of optical filters.
Polymer (Plastics)^67^	130	400–1600	~1.5	Inexpensive but typically higher CTE and lower mechanical strength, and environmental resilience.
Sapphire (Al_2_O_3_)^68^	4.0	150–6000	~1.8	High transparency. High CTE, chemically inert. High cost and limited wafer sizes. Difficult to etch.

The input objects shown in Fig. 6a were fabricated on a glass slide using EBL and a metal lift-off process. First, the e-beam resist polymethyl methacrylate (PMMA) was spin-coated onto a glass slide and baked on a hot plate at 180 °C for 5 min. After e-beam exposure, the resist was developed in a 3:1 solution of methyl isobutyl ketone (MIBK) and isopropyl alcohol (IPA) for 90 s, followed by a rinse in IPA for 30 s. Subsequently, a 100 nm layer of aluminum was deposited on the sample via magnetron sputtering. The resist was then removed using N-Methyl-2-pyrrolidone (NMP) in an ultrasonic bath at 80 °C. Finally, after the lift-off process, the sample was rinsed for surface cleaning with IPA. This method allowed us to create hundreds of binary amplitude input objects on a single slide.

### Experimental setup

The experimental setup shown in Fig. 5 incorporated a supercontinuum laser light source (WhiteLase-Micro; Fianium Ltd, Southampton, UK) to provide multispectral illumination, a microscope slide holder (MAX3SLH; Thorlabs, Inc.) equipped with a sample clip to securely hold the input object slide, and a 3D positioning stage (MAX606; Thorlabs, Inc.) to enable alignment of the object slide. The output power of the supercontinuum light source varied across different wavelengths, as shown in Fig. S9. A color CMOS image sensor chip (16.4 MP resolution, 1.12 μm pixel size, Sony Corp., Japan) was employed to capture the image patterns, and a PC controlled the operations of the entire setup. The 3D positioning stage provided precise control over the 500 μm separation between the sample slide and the first diffractive layer, as well as lateral adjustments to position different input objects on the slide. A custom-designed holder, fabricated with a 3D printer (Objet30 Pro, Stratasys), stabilized the diffractive design and maintained a 500 μm separation between the second diffractive layer and the sensor.

In the experimental demonstration of unidirectional visible imaging, we simultaneously illuminated the sample with three distinct wavelengths to achieve multispectral imaging. To address the spectral crosstalk errors among the color channels in the RGB image sensor, we applied a demosaicing algorithm^69,70^. This crosstalk correction is computed by the following equation:

13 $$\begin{array}{c}\left[\begin{array}{c}{U}_{R}\\ {U}_{G}\\ {U}_{B}\end{array}\right]={\bf{W}}\times \left[\begin{array}{c}\begin{array}{c}{U}_{R{{\_}}{ori}}\,\\ {U}_{{G}_{1}{{\_}}{ori}}\end{array}\\ {U}_{{G}_{2}{{\_}}{ori}}\\ {U}_{B{{\_}}{ori}}\end{array}\right]\end{array}$$

Here, ${U}_{{R\_ori}}$, ${U}_{{G}_{1}{\_ori}}$, ${U}_{{G}_{2}{\_ori}}$ and ${U}_{{B\_ori}}$ denote the original patterns captured by the image sensor, **W** represents a 3 × 4 crosstalk matrix obtained through experimental calibration specific to the RGB sensor chip, and ${U}_{R}$, ${U}_{G}$, and ${U}_{B}$ are the corrected (R, G, B) image patterns.

## Supplementary information

Supplementary Information

## Data Availability

All the data and methods needed to evaluate the conclusions of this work are presented in the main text and Supplementary Information. Additional data can be requested from the corresponding author.

## Code availability

The codes used in this work use standard libraries and scripts that are publicly available in PyTorch (Meta).
