# Supplementary material for: Broadband unidirectional visible imaging using wafer-scale nano-fabrication of multi-layer diffractive optical processors
Source: Light Sci Appl. 2025 Aug 11;14:267. doi: 10.1038/s41377-025-01971-2 (PMC12340160; doi:10.1038/s41377-025-01971-2)
Supplement: Supplementary file 1 — Supplementary Information [file 41377_2025_1971_MOESM1_ESM.pdf]

Supplementary Information for

Broadband Unidirectional Visible Imaging Using Wafer-Scale Nano-Fabrication of Multi-Layer Diffractive Optical Processors

Che-Yung Shen[1,2,3], Paolo Batoni[4], Xilin Yang[1,2,3], Jingxi Li[1,2,3], Kun Liao[1], Jared Stack[4], Jeff Gardner[4], Kevin Welch[4] and Aydogan Ozcan[1,2,3*]

[1]Electrical and Computer Engineering Department, University of California, Los Angeles, CA, 90095, USA

[2]Bioengineering Department, University of California, Los Angeles, CA, 90095, USA

[3]California NanoSystems Institute (CNSI), University of California, Los Angeles, CA, 90095, USA

[4]Micro Optics, Optical Systems Division, Broadcom Inc., Charlotte, NC, 28262, USA

[*]Correspondence to: ozcan@ucla.edu

Contents:

Figure S1. Thickness profiles of the diffractive layers corresponding to the three-layer unidirectional imager design.

Figure S2. The trade-off between the broadband unidirectional imaging performance and the output diffraction efficiencies of diffractive imagers.

Figure S3. External generalization of the broadband unidirectional visible imager on randomly shifted objects.

Figure S4. External generalization of the broadband unidirectional visible imager on randomly rotated objects.

Figure S5. External generalization of the broadband unidirectional visible imager on randomly scaled objects.

Figure S6. Confocal microscopy images of the fabricated diffractive layers.

Figure S7. Experimental results of broadband unidirectional imaging under different illumination wavelengths.

Figure S8. Refractive index of the high purity fused silica as a function of wavelength.

Figure S9. Output power distribution of the laser light source as a function of the wavelength.

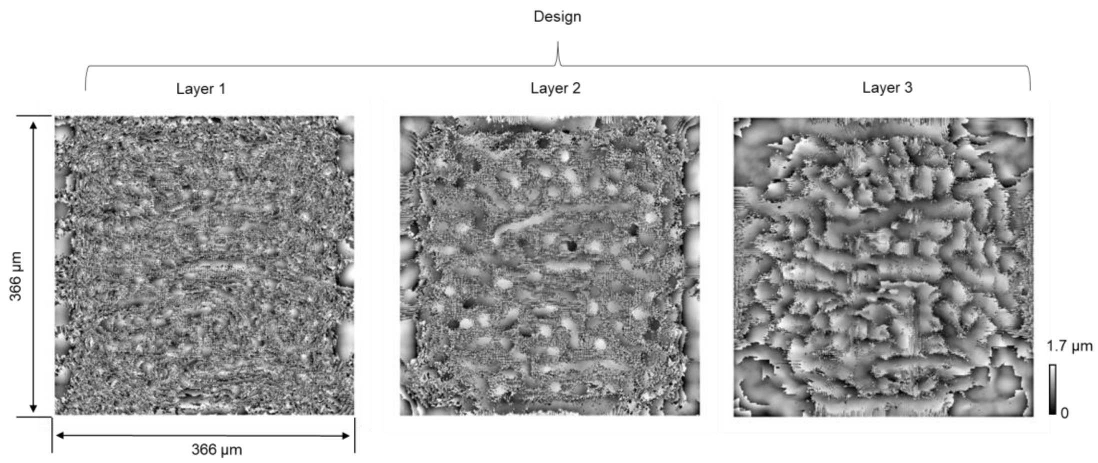

Figure S1. Thickness profiles of the diffractive layers corresponding to the three-layer unidirectional imager design.

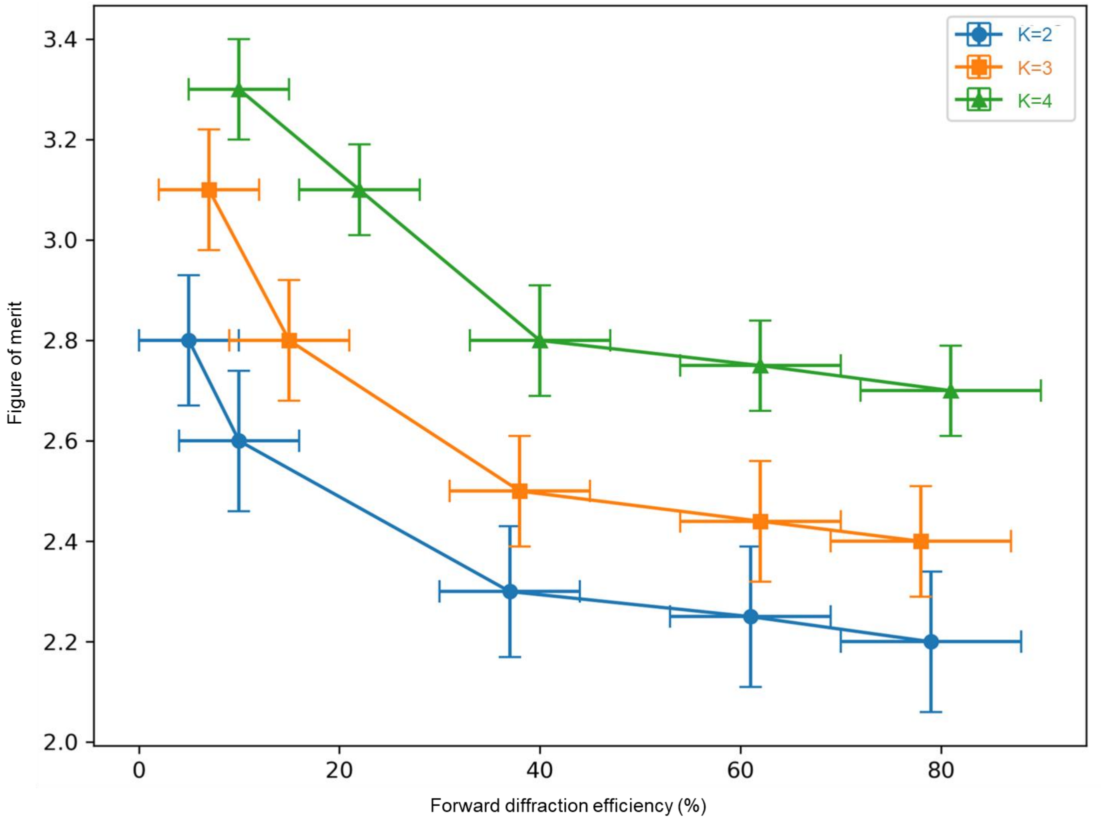

Figure S2. The trade-off between the broadband unidirectional imaging performance and the output diffraction efficiencies of diffractive imagers. The figure of merit (FOM) on the y-axis reflects the mean value computed over the 5000 test objects from the MNIST image dataset. The data points correspond to diffractive imagers trained with a diffraction efficiency-related penalty term, as defined in Eq. (12) of the main text. Three configurations—two-layer (K=2), three-layer (K=3), and four-layer (K=4) diffractive processors—were evaluated under varying forward diffraction efficiency weights (0.0001, 0.0003, 0.0006, 0.0009, 0.0012) corresponding to the data points from left to right on the plot. Crosses in the plot indicate the standard deviations of the forward diffraction efficiencies and the FOM values for different unidirectional imager designs.

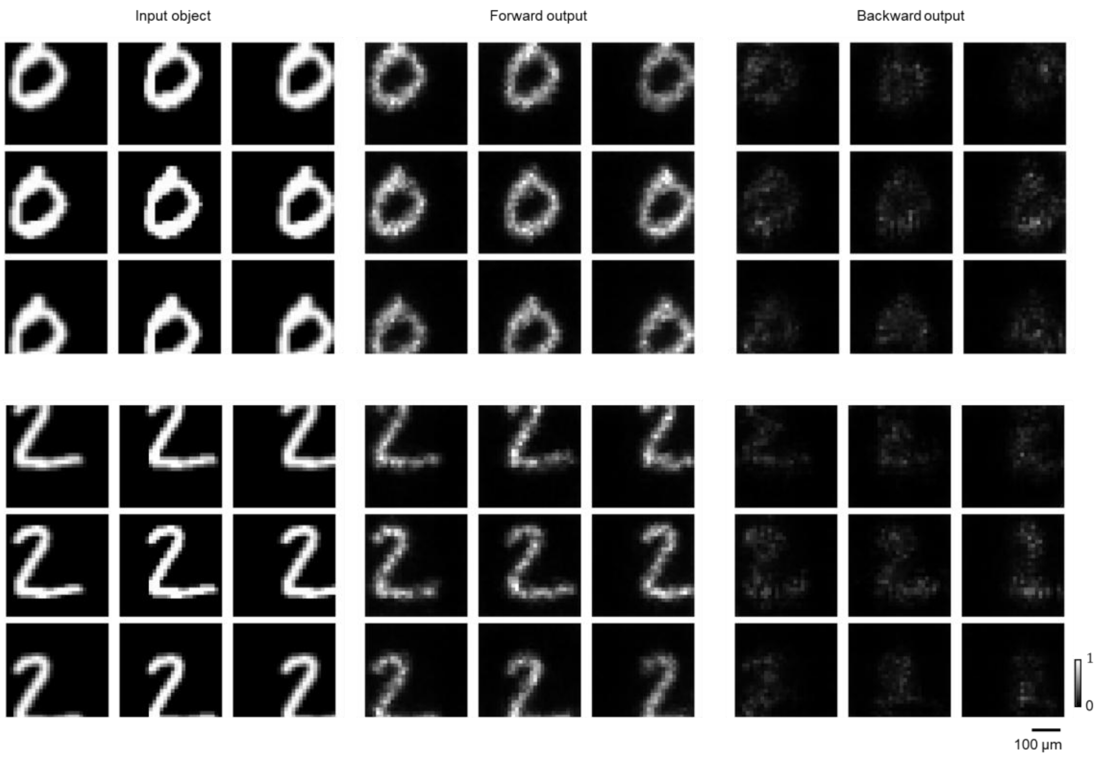

Figure S3. External generalization of the broadband unidirectional visible imager on randomly shifted objects. Output image examples of both the forward and backward directions, along with the input objects at different positions.

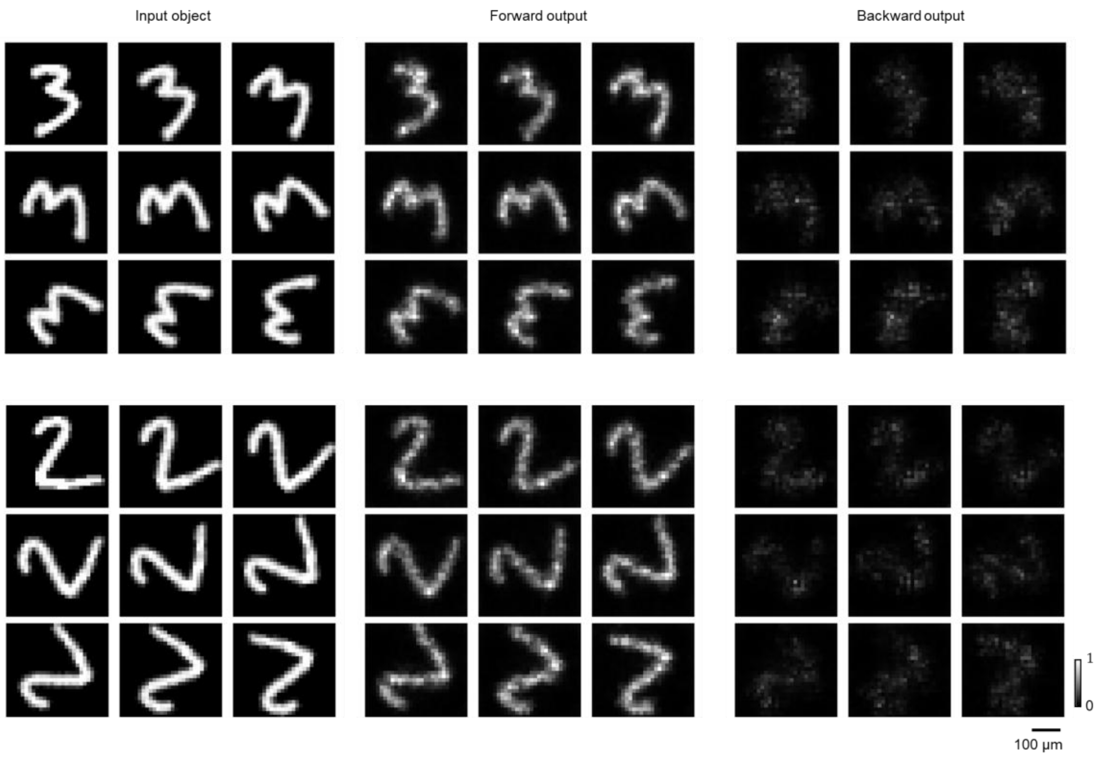

Figure S4. External generalization of the broadband unidirectional visible imager on randomly rotated objects. Output image examples of both the forward and backward directions, along with the input objects with different orientations.

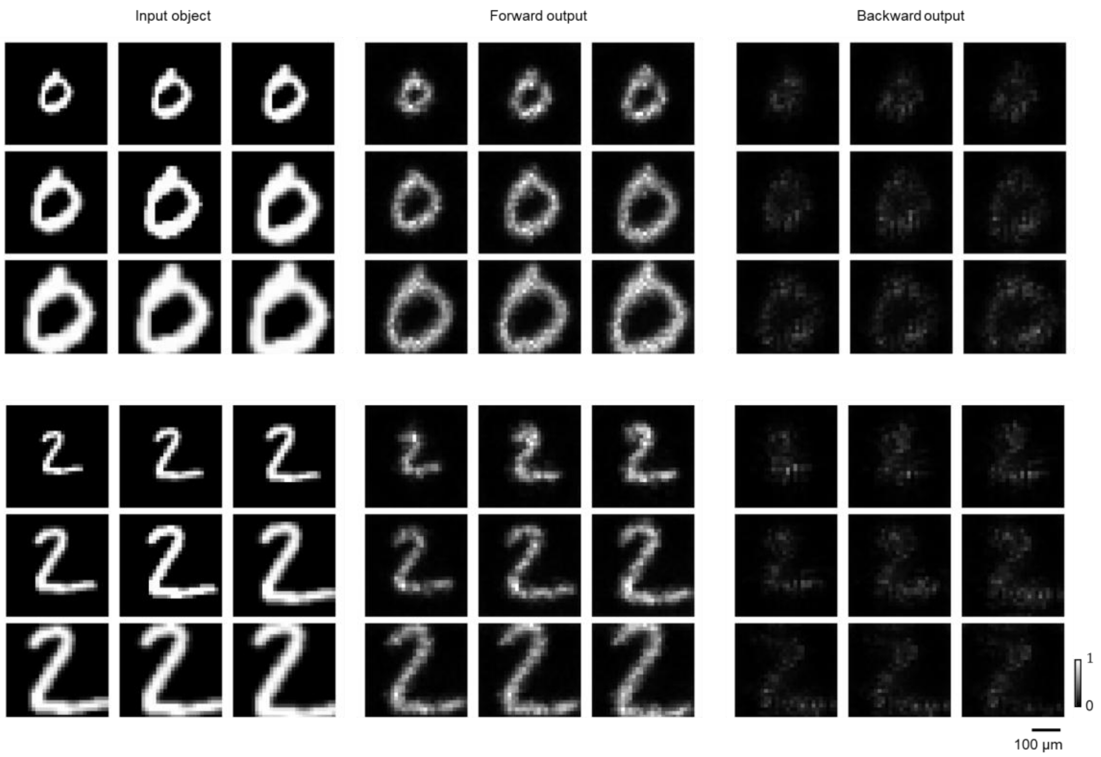

Figure S5. External generalization of the broadband unidirectional visible imager on randomly scaled objects. Output image examples of both the forward and backward directions, along with the input objects at different scales.

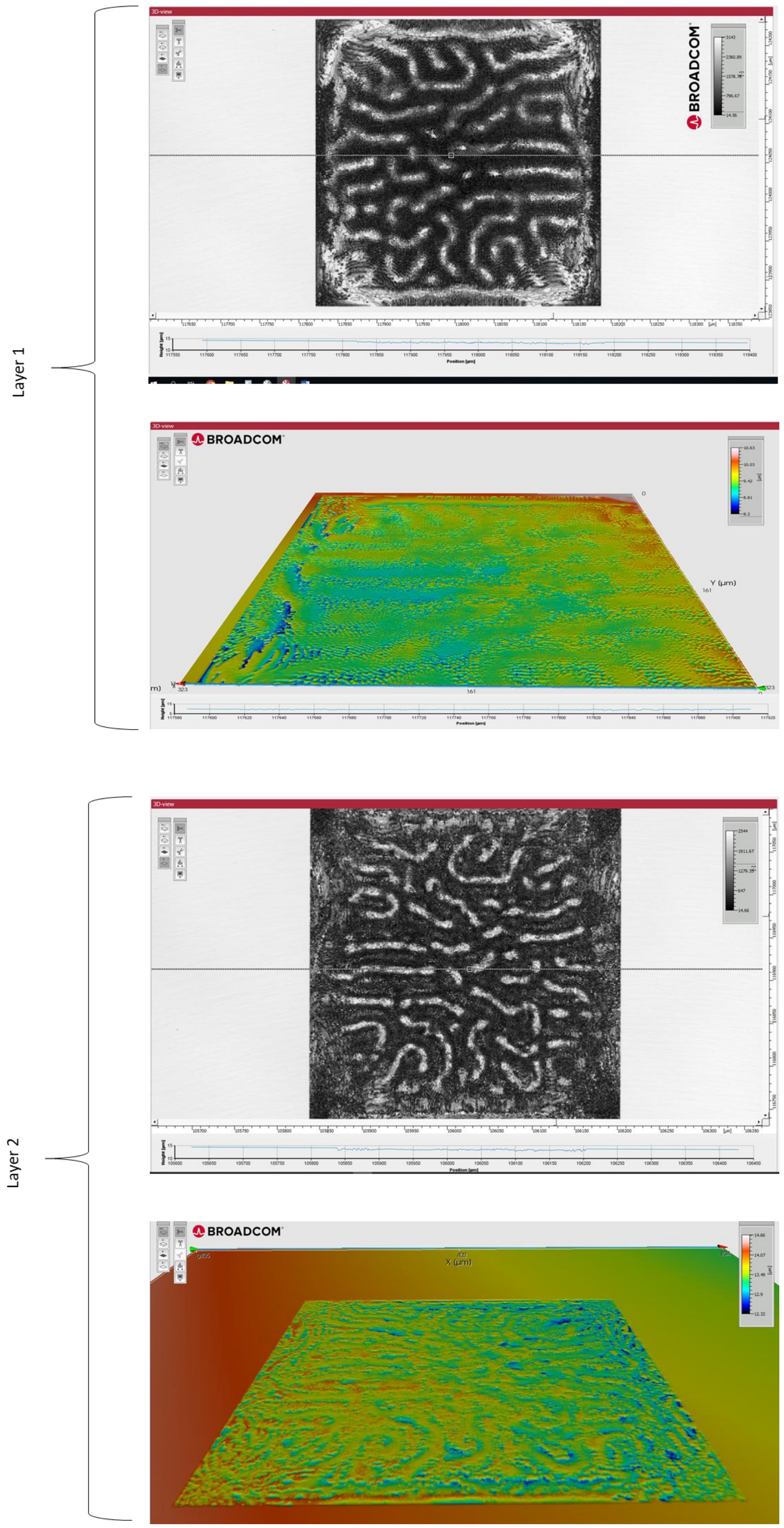

Figure S6. Confocal microscopy images of the fabricated diffractive layers.

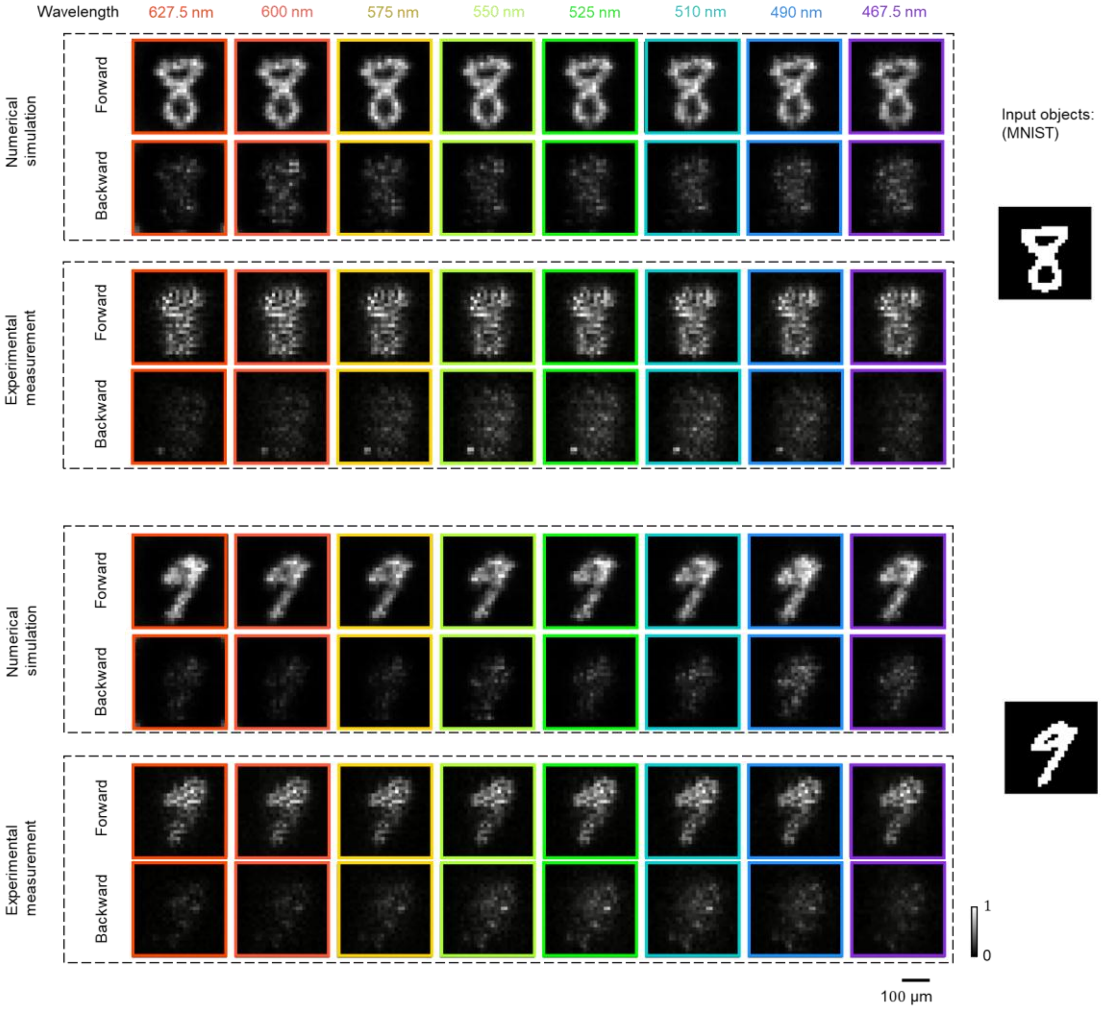

Figure S7. Experimental results of broadband unidirectional imaging under different illumination wavelengths. Experimentally measured diffractive output images of both the forward and the backward directions at different wavelengths. The color frame surrounding each image corresponds to the visible color associated with the respective illumination wavelength.

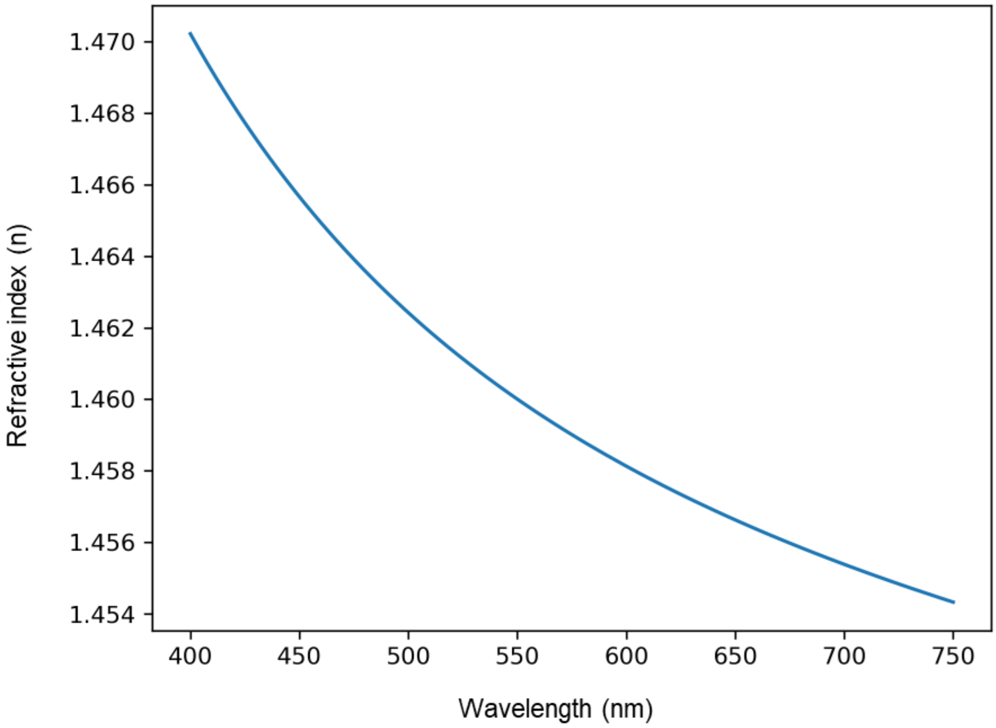

Figure S8. Refractive index of the high purity fused silica as a function of wavelength.

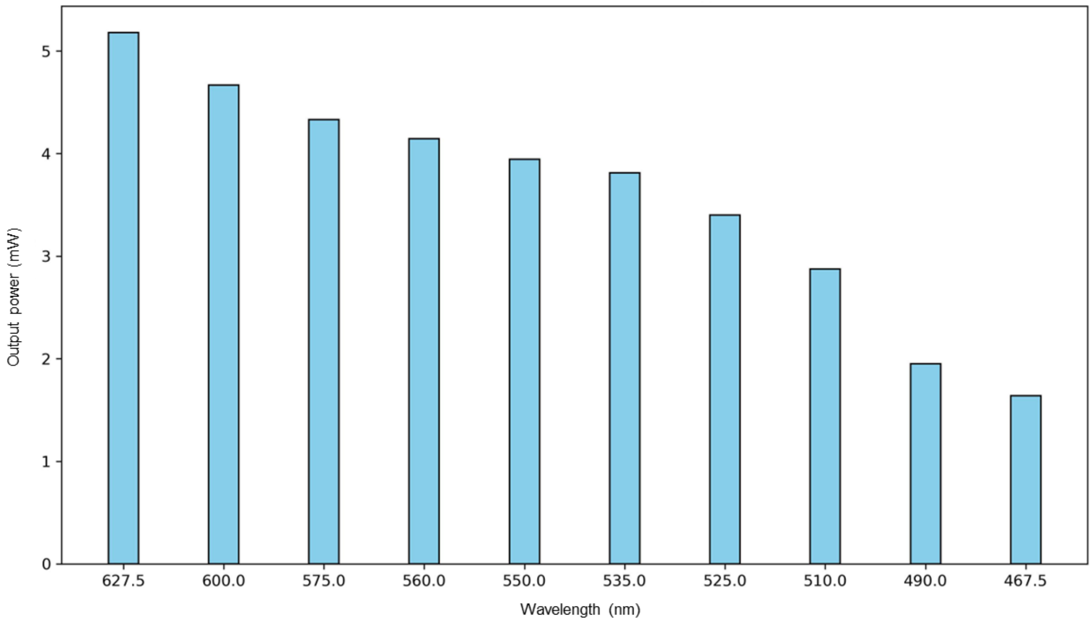

Figure S9. Output power distribution of the laser light source as a function of the wavelength.
